# 3D predictability assessment of clear aligners versus fixed appliances using CBCT

**DOI:** 10.6026/973206300222219

**Published:** 2026-04-30

**Authors:** Subash Chandra Nayak, Madhu Bhaskar Bommasani, Uday Sagar Maddula N.V, Charan Teja V.V, Siddardha S.J, Vijay Srikrishna Varma Rudraraju, Rubeena Naaz, Md Kafeel Ahmed

**Affiliations:** 1 Department of Orthodontics and Dentofacial Orthopaedics, Hi-Tech Dental College and Hospital, Bhubaneshwar, Odisha, India; 2 Department of Orthodontics and Dentofacial Orthopaedics, St. Joseph Dental College & Hospital, Duggirala, Eluru, Andhra Pradesh, India; 3 Private Practitioner, Dental Surgeon, SRK Dental Clinic, Hyderabad, Telangana, India; 4 Department of Periodontology and Implantology, MNR Dental College and Hospital, Sangareddy, Hyderabad, Telangana, India

**Keywords:** Clear aligners, fixed appliances, cone-beam computer tomography (CBCT), three-dimensional analysis, orthodontic predictability, tooth movement, treatment planning

## Abstract

Predictability of orthodontic tooth movement varies between clear aligners and fixed appliances, yet three-dimensional comparative evidence remains limited for complex cases. This prospective study randomized 60 complex orthodontic patients to clear aligners (n=30) or fixed appliances (n=30), using baseline/planned/post-treatment CBCT superimposition to quantify accuracy across three planes of space. Clear aligners excelled in incisor torque (87.3% versus 82.2%, p=0.001) and anterior intrusion (91.2% versus 82.8%, p=0.002), while fixed appliances were superior for canine/premolar rotation (88.9% versus 78.6%, p=0.001) and anterior extrusion (85.7% versus 76.2%, p=0.004). Overall treatment predictability was comparable (clear aligners 84.6±7.8% versus fixed 82.3±8.9%, p=0.286), revealing modality-specific strengths for targeted movements. This data help in orthodontic planning by establishing CBCT-based evidence for modality selection based on movement type, enhancing precision in complex cases.

## Background:

Modern orthodontics has been characterised by the rapid adoption of technology, with a new technique, clear aligner therapy, introduced to replace conventional fixed appliances, thereby entirely changing treatment methods and patient expectations [1]. Clear aligners, which are made using sequential removable devices manufactured from a computer-generated model, have proven widely acceptable to both patients and practitioners because of their aesthetics, improved oral health maintenance and greater patient comfort during treatment [2]. Predictability of planned tooth movements is, however, a major consideration in planning/treatment and outcome assessment, given that the complexity of cases addressed with clear aligners continues to increase [3]. Over many decades of clinical experience and research, traditional fixed appliance therapy has proven consistent and predictable, delivering a multitude of orthodontic movements [4]. The biomechanical concepts of fixed appliance therapy have been well developed, with a large body of literature on the effectiveness of a wide range of wire sequences, bracket prescriptions and auxiliary mechanics to produce the required tooth movements [5]. The three-dimensional treatment predictability analysis, however, has been curtailed by the traditional two-dimensional radiographic analysis, which does not capture the complexity of orthodontic tooth movement across all spatial dimensions [6]. The introduction of cone-beam computed tomography (CBCT) has transformed orthodontic diagnosis and treatment planning because this technique can provide high-resolution three-dimensional images and low radiation doses compared with traditional computed tomography [7]. CBCT technology enables in-depth evaluation of tooth position, root morphology and anatomical structures, thereby improving treatment planning and treatment outcomes [8].

The combination of CBCT data with computer-aided design and production technologies has further improved the accuracy of orthodontic treatment planning, specifically in clear aligner therapy, where virtual treatment plans direct the fabrication of appliances [9]. Recent studies examining the predictability of clear aligners have shown inconsistent accuracy across different types of tooth movements, with generally better predictability for simple tipping movements and lower predictability for complex ones such as rotation, torque and vertical displacement [10]. Studies have shown that clear aligners achieve about 41 per cent of predetermined rotational movements with cylindrically shaped teeth and 47 per cent with root-shaped teeth, indicating that their rotational predictability is greatly restricted [11]. On the same note, torque movements, especially those of the anterior region, are variably predictable with clear aligners, with some studies reporting accuracy rates of 42 to 78 percent, depending on the magnitude and direction of intended motion [12]. The outcomes of treatment and patient satisfaction have been the most common comparative studies performed between clear aligners and fixed appliances, compared with a limited number of studies on the predictability of movement using three-dimensional models [13]. The majority of studies have used two-dimensional analysis or indirect measurement techniques, which may not accurately indicate the complications of orthodontic tooth movement [14]. Other small-sample studies that use three- dimensional analysis have been limited by a lack of uniform treatment goals or by a focus on single-tooth movement rather than the entire assessment of the tooth [15]. The clinical impact of learning predictability variations across treatment modalities is not limited to the academic community but has direct consequences for treatment planning choices, patient counseling and treatment duration estimates [16]. Practitioners need evidence-based data on the comparative advantages and disadvantages of each treatment modality to maximize treatment selection in individual patients and to meet particular movement demands [17]. Moreover, the growing complexity of cases treated with clear aligners requires in-depth knowledge of movement constraints to avoid complications and achieve the best outcomes [1]. Therefore, it is of interest to assess and compare the predictability of clear aligners and fixed appliances in producing intended orthodontic tooth movements with the aid of the CBCT analysis. This will help to determine the predictability of various types of movements, different regions of the body and the level of complexity of the given treatment.

## Materials and Methods:

## Design of the study and ethics approval:

It was a prospective, randomized, parallel-group comparative study conducted at the Department of Orthodontics, University Hospital, between January 2022 and December 2023.

## Sample size calculation:

The sample size was determined based on anticipating variation in the overall predictability of treatment between clear aligners and fixed appliances. Given a population difference in predictability of 8 percent, a standard deviation of 12 percent, an alpha error of 0.05 and a power of 80, a minimum of 26 patients per group was necessary. The expected dropout rates necessitated recruiting 30 patients in each treatment group to enable subgroup analyses.

## Participant selection:

Sixty patients who requested orthodontic treatment were recruited based on present inclusion and exclusion criteria. The inclusion criteria included patients aged 14-35 years with permanent dentition, Class I or mild Class II malocclusion requiring complete treatment, without severe periodontal disease and willing to adhere to treatment procedures and follow-up. Patients volunteered and maintained good oral hygiene, with a plaque index of less than 20% among the total respondents. The exclusion criteria were severe skeletal discrepancies that necessitated orthognathic surgery, severe periodontal disease with alveolar bone loss of more than 30 percent root length, prior orthodontic treatment, missing teeth (except third molars), much restorative work (more than 25 percent clinical crowns), temporomandibular joint disorders, pregnancy and systemic diseases that altered bone metabolism or wound healing.

## Randomization and allocation of treatment:

Eligible patients were randomly assigned to a control condition of either clear aligner therapy or fixed appliance therapy using computer- generated random sequences in sealed, non-transparent envelopes. Randomization of blocks of different sizes was used to maintain balance during recruitment. The allocation of treatment was made upon completion of the initial records and treatment arrangements.

## Treatment planning and implementation:

Every patient was thoroughly examined using orthodontic procedures, including clinical examination, dental impressions, photos and CBCT. Experienced orthodontists developed treatment plans in both modalities and when feasible, the objectives of movements were standardized between groups. Crystal aligner patients were treated with a commercially available system (Invisalign, Align Technology), whereas fixed appliances were treated with pre-adjusted edgewise brackets with a 0.022-inch slot.

## CBCT Imaging protocol:

They were acquired as CBCT scans on a large-field-of-view scanner (voxel size 0.3 mm, 120 kVp, 8 mA, scan time 26.9 seconds) using a standardized protocol. The patients were placed in the Franklin horizontal position parallel to the floor and instructed to maintain centric occlusion during scanning. Each patient underwent three CBCT scans: baseline (T0), treatment-planning phase (T1) and treatment completion (T2).

## Three-Dimensional measurement and analysis:

The CBCT data were imported into orthodontic analysis software (Dolphin Imaging and Management Solutions) to analyze them three- dimensionally. The stable anatomical landmarks, such as the anterior cranial base of the maxillary measures and the mandibular measures of the canal and symphysis, were used for automated registration and superimposition. Changes in individual tooth positions were measured using the positional distance between planned and achieved positions in each of the three planes of space.

## Predictability assessment:

Treatment predictability was determined as the percentage of intended movement achieved and a value near 100 percent indicated greater predictability. Measurements were done on linear (mesial-distal, buccal-lingual, intrusion-extrusion) and angular (tip, torque, rotation) movements on all teeth of the first molar-first molar in each arch. Direction, magnitude and anatomical location were used to classify movements and provide an adequate examination.

## Clinical monitoring and compliance assessment:

Monitoring was conducted in a standardized manner, one after another: every 4-6 weeks for clear aligners and every 6-8 weeks for fixed appliances. Compliance in both groups was measured by aligner tracking (for clear aligner patients) and appointment attendance. The alterations and improvements in treatment were documented and included in the final predictability calculations.

## Statistical analysis:

The statistical test was conducted using SPSS version 28.0. Continuous and categorical variables were reported with descriptive statistics, including means, standard deviations and percentages, respectively. The Shapiro-Wilk test was used to assess the distribution's normality. Independent t-tests were used to compare groups on normally distributed continuous variables and the Mann-Whitney U test was used to compare groups on non-normally distributed continuous variables. Categorical variables were tested using Chi-square tests. Multiple group comparisons were performed using analysis of variance (ANOVA) and pairwise comparisons were performed using the post-hoc Tukey tests. Linear regression was carried out to identify predictors of treatment predictability. All analyses were established at p < 0.05.

## Results:

Sixty patients completed the study protocol, with 30 patients in each treatment group. No patients were lost to follow-up during the study period. Baseline demographic characteristics were well-balanced between groups, with mean ages of 22.4 ± 6.8 years for clear aligner patients and 23.1 ± 7.2 years for fixed appliance patients (p = 0.681). Treatment duration averaged 18.6 ± 4.2 months for clear aligners and 20.8 ± 5.1 months for fixed appliances (p = 0.086) (Table 1 - see PDF). Overall treatment predictability was comparable between clear aligners and fixed appliances (84.6 ± 7.8% versus 82.3 ± 8.9%, p = 0.286). However, significant differences were observed for specific movement types and anatomical regions. Clear aligners demonstrated superior predictability for anterior tooth movements, while fixed appliances showed better performance for posterior tooth movements (Table 2 - see PDF). Analysis by movement type revealed distinct patterns of predictability between treatment modalities. Clear aligners showed significantly higher accuracy in controlling incisor torque and intrusion movements, while fixed appliances demonstrated superior predictability for rotational movements and posterior tooth displacement. Anatomical region analysis revealed heterogeneous predictability patterns, with anterior teeth exhibiting higher overall predictability than posterior teeth in both treatment groups. Maxillary teeth generally exhibited better predictability than mandibular teeth, particularly for complex movements (Table 3 - see PDF). Linear regression analysis revealed significant correlations between treatment complexity and predictability outcomes. Cases requiring greater than 4mm of space closure demonstrated reduced predictability in both treatment groups (clear aligners: r = -0.68, p < 0.001; fixed appliances: r = -0.43, p = 0.018). Similarly, cases with extensive rotational requirements (>15 degrees per tooth) showed decreased accuracy, particularly in the clear aligner group. Patient compliance significantly influenced treatment predictability in the clear aligner group (r = 0.72, p < 0.001) but showed minimal correlation in the fixed appliance group (r = 0.31, p = 0.092). Clear aligner patients with compliance rates below 80% demonstrated markedly reduced predictability across all movement types (76.2 ± 11.8% versus 89.4 ± 6.7%, p < 0.001).

## Discussion:

This three-dimensional examination provides new insight into the relative predictability of clear aligners and a fixed appliance under different orthodontic movements. The observation that the overall predictability of treatment was similar across modalities (84.6 versus 82.3) contravenes traditional beliefs regarding the superiority of one treatment modality over another. It supports the idea that both systems can be used to achieve predictable outcomes under the right circumstances [9]. Nonetheless, the wide variations in the predictability of movements necessitate selecting the treatment modality based on the desired tooth movements, rather than on universal preferences. The better forecast ability of clear aligners for incisor torque regulation (87.3% compared to 76.4%) is a clinically significant observation that conflicts with some earlier investigations indicating restrictions in torque expression with aligners [11, 12, 13- 14]. Such enhanced torque predictability can be explained by the fact that the aligners provide full crown coverage, allowing more efficient application of forces to achieve root movement compared to bracket-based systems. The three- dimensional analysis methodology used in the current study can also provide a more precise evaluation of torque movements than the customary two-dimensional evaluation techniques applied in previous studies. The higher rotational movement performance of fixed appliances, especially in the canine and premolar areas (88.9 versus 78.6 and 87.1 versus 75.2), on the other hand, does not contradict current biomechanical principles or clinical observations [2]. The application of point-contact forces and accurate bracket positioning in the fixed appliance system provides the mechanical advantages of rotational movement. They are difficult to reproduce with aligner therapy. These results support a clinical suggestion that fixed appliance therapy should be used in instances requiring a large number of rotational adjustments, especially in cylindrical-shaped teeth, where attachment placement can be difficult. The predictability difference in the region: the anterior teeth demonstrate better performance than the posterior teeth in both modalities, which is indicative of the biomechanical difficulties of moving larger, multi-rooted teeth [3]. The lower predictability in the posterior parts, indicating the clear aligner therapy (79.2% versus 86.1% maxillary, 77.8% versus 84.3% mandibular), may indicate an inability to deliver and retain force in the anterior parts. The implications of these findings for treatment planning include that significant posterior tooth movement or space closure necessitates their use. Patient compliance and the predictability of treatment in clear aligner therapy (r = 0.72) are strongly correlated, underscoring the paramount role of treating patients and motivating them to achieve the best results [17, 18]. This was something that was quite lacking in fixed appliance therapy, thus illustrating the core difference in the mechanisms of delivering treatment across modalities. The observation that compliance below 80 percent led to a significant decrease in predictability (76.2% versus 89.4%) provides objective information on setting minimum compliance rates during clear aligner treatment.

## Conclusion:

CBCT analysis reveals comparable overall predictability between clear aligners and fixed appliances. However, but clear aligners excel in incisor torque/intrusion and fixed appliances dominate rotation/posterior displacement. Treatment modality selection should prioritize specific movement requirements over general preference, with patient compliance critically influencing aligner success. This data help improve orthodontic precision through 3D analysis, emphasizing case selection and stratification by complexity for optimal outcomes in both systems.
